# Chemical screening for promotion of adventitious root formation in *Eucalyptus globulus*

**DOI:** 10.1186/1753-6561-5-S7-P139

**Published:** 2011-09-13

**Authors:** Naoki Negishi, Masatoshi Oishi, Akiyoshi Kawaoka

**Affiliations:** 1 Nippon Paper Industries Co., LTD., Agri-Biotechnology Research Laboratory

## 

*Eucalyptus globulus*is one of the most profitable trees for pulp and paper industries due to its fast growth and short harvesting cycle. This species is easily pulped with high yield, and its fiber qualities are among the best for paper production. However, it is difficult to vegetatively propagate *E. globulus*.So far, we have developed “photoautotropic culture method” that promotes rooting percentage by feeding approximately three times higher level CO_2_ level (1000 µmol mol^–1^) and suitable culture condition [1]. However, several lines showed poor rooting percentage even in the higher CO_2_ conditions. Therefore, it was necessary to develop novel method for promotion of adventitious root (AR) formation in *E. globulus*.

First, we measured endogenous levels of 20 kinds of hormones such as abscisic acid, auxins, cytokinines and gibberellins, at basal part of stem of easy-rooting line and poor-rooting line by UPLC-ESI-qMS/MS. As a result, the Indole-3-acetic acid (IAA) level of easy-rooting line was two times higher than that of the poor-rooting line, suggesting that endogenous IAA level may regulate ability of AR formation.

Next, we focused on the cytochrome P450s that are involved in a vast array of reactions of many different metabolic pathways. Several triazole-containing chemical compounds have previously been shown act as efficient inhibitors of cytochrome P450 monooxygenases. A chemical library of triazole derivatives to find chemicals which have the effect of promoting AR formation was screened. Consequently, five compounds effectively promoted AR formation.

Finally, we investigated how these chemicals affected the growth of *Arabidopsis thaliana*. *Arabidopsis* seedlings were grown on agar medium containing 1 μM selected chemicals. One of the selected chemicals, MA65 increased the number of roots in wild-type *Arabidopsis* seedlings and this phenotype was similar to a mutant *superroot2* (*sur2*) [2]. The *SUR2* gene encodes the cytochrome P450 CYP83B1, a modulator of auxin homeostasis. The amounts of endogenous IAA in 14-d-old Arabidopsis grown in the presence of 1 mM MA65 were analyzed. The IAA content was increased two-fold in the presence of MA65 as compared with untreated* Arabidopsis*. In addition, 1.0 cm explants of Arabidopsis stems were incubated for 7 d on MS medium containing 1 mMMA65. Stimulation of an AR formation was observed as compared to untreated samples. Taken together, MA65 may increase endogenous IAA level in a plant cell and promotes an AR formation.
